# Evaluation of elastix-based propagated align algorithm for VOI- and voxel-based analysis of longitudinal ^18^F-FDG PET/CT data from patients with non-small cell lung cancer (NSCLC)

**DOI:** 10.1186/s13550-015-0089-z

**Published:** 2015-03-21

**Authors:** Gerald SMA Kerner, Alexander Fischer, Michel JB Koole, Jan Pruim, Harry JM Groen

**Affiliations:** University of Groningen and Department of Pulmonary Diseases, University Medical Center Groningen, Hanzeplein 1, P.O. Box 30.001, , 9700 RB Groningen, The Netherlands; Philips Technologie GmbH Innovative Technologies, Postfach 40, Philipstr. 8, Aachen, 52068 Germany; University of Groningen and Department of Nuclear Medicine and Molecular Imaging, University Medical Center Groningen, Hanzeplein 1, P.O. Box 30.001, , 9700 RB Groningen, The Netherlands; Department of Nuclear Medicine, Faculty of Medicine & Health Sciences, Tygerberg Hospital, Stellenbosch University, Francie van Zijl drive, Cape Town, 7505 South-Africa

**Keywords:** Elastix, PET/CT, Image deformation, NSCLC

## Abstract

**Background:**

Deformable image registration allows volume of interest (VOI)- and voxel-based analysis of longitudinal changes in fluorodeoxyglucose (FDG) tumor uptake in patients with non-small cell lung cancer (NSCLC). This study evaluates the performance of the elastix toolbox deformable image registration algorithm for VOI and voxel-wise assessment of longitudinal variations in FDG tumor uptake in NSCLC patients.

**Methods:**

Evaluation of the elastix toolbox was performed using ^18^F-FDG PET/CT at baseline and after 2 cycles of therapy (follow-up) data in advanced NSCLC patients. The elastix toolbox, an integrated part of the IMALYTICS workstation, was used to apply a CT-based non-linear image registration of follow-up PET/CT data using the baseline PET/CT data as reference. Lesion statistics were compared to assess the impact on therapy response assessment. Next, CT-based deformable image registration was performed anew on the deformed follow-up PET/CT data using the original follow-up PET/CT data as reference, yielding a realigned follow-up PET dataset. Performance was evaluated by determining the correlation coefficient between original and realigned follow-up PET datasets. The intra- and extra-thoracic tumors were automatically delineated on the original PET using a 41% of maximum standardized uptake value (SUV_max_) adaptive threshold. Equivalence between reference and realigned images was tested (determining 95% range of the difference) and estimating the percentage of voxel values that fell within that range.

**Results:**

Thirty-nine patients with 191 tumor lesions were included. In 37/39 and 12/39 patients, respectively, thoracic and non-thoracic lesions were evaluable for response assessment. Using the EORTC/SUV_max_-based criteria, 5/37 patients had a discordant response of thoracic, and 2/12 a discordant response of non-thoracic lesions between the reference and the realigned image. FDG uptake values of corresponding tumor voxels in the original and realigned reference PET correlated well (*R*^2^=0.98). Using equivalence testing, 94% of all the voxel values fell within the 95% range of the difference between original and realigned reference PET.

**Conclusions:**

The elastix toolbox impacts lesion statistics and therefore therapy response assessment in a clinically significant way. The elastix toolbox is therefore not applicable in its current form and/or standard settings for PET response evaluation. Further optimization and validation of this technique is necessary prior to clinical implementation.

**Electronic supplementary material:**

The online version of this article (doi:10.1186/s13550-015-0089-z) contains supplementary material, which is available to authorized users.

## Background

Tumor response during or after treatment is an important surrogate marker for survival in oncological patients. Most commonly, tumor response is measured with CT using predefined anatomical criteria such as the Response Evaluation Criteria in Solid Tumors (RECIST) criteria or volumetric criteria [1]. In the past years, metabolic response assessment with ^18^ F-FDG PET has gained importance for patients treated with chemotherapy. Especially for patients who are treated with tyrosine kinase inhibitors, metabolic lesion changes as measured with PET have been proven to precede lesion size alterations measurable with CT [2-4]. In order to standardize metabolic response assessment with ^18^ F-FDG PET, the European Organization for Research and Treatment of Cancer (EORTC) recommendations and more recently the PERCIST criteria were drafted as guidance for clinical practice [5,6].

Traditionally, ^18^ F-FDG lesion uptake is quantified by the maximum and/or mean standardized uptake value (SUV) of tumor lesions. Although these measures (specifically maximum standardized uptake value (SUV_max_)) have a prognostic value in a variety of cancers, including non-small cell lung cancer (NSCLC) [7-10], flaws in SUV quantification can be introduced by a variety of factors including the type of equipment used, differences in PET acquisition protocols, reconstruction parameters, analysis procedures, and image statistics. Moreover, textural changes in tumor heterogeneity better reflect tumor response and survival than SUV quantification alone [11].

Consequently, research efforts have attempted to improve the predictive nature of PET by taking into account all voxel values and using voxel-wise approaches for image analysis. For other imaging modalities, such as MR imaging, a so-called voxel-by-voxel analysis is well established. It has been proven to be a viable technique for measuring therapy response in breast cancer [12], while for high-grade glioma, it was predictive for the overall survival rate [13]. Voxel-based analysis has also been applied to response assessment with PET images in head and neck cancer patients and was shown to be a useful tool [14].

Deformable image registration may help to improve tumor response assessment. The elastix toolbox is a modular computer program for intensity-based medical image registration. The elastix toolbox [15] has been validated for thoracic deformable CT to CT image registration demonstrating a good overall performance compared to other available algorithms [16]. Theoretically, this algorithm can also be applied to the PET/CT datasets using the corresponding sequentially acquired low-dose CT data. Because of the public domain nature of the program, the modular adaptability, and the previous good performance, the toolbox has been integrated with the propagated align algorithm to perform PET/CT to PET/CT deformable image registration. To the best of our knowledge, the performance of the elastix toolbox on the alignment of solid lung tumors or the impact on intratumoral distribution of ^18^ F-FDG uptake has not been assessed so far. The aim of this study is to evaluate the applicability of the elastix deformable registration for a volume of interest (VOI)-based evaluation of lesion statistics.

## Methods

Baseline VOIs and transformed and interpolated follow-up data and a voxel-by-voxel longitudinal mapping of ^18^ F-FDG uptake in a clinical setting of patients with advanced NSCLC were used. For this evaluation, the different steps necessary to perform PET/CT to PET/CT alignment with the elastix toolbox were integrated in the IMALYTICS Research Workstation to make the CT-based deformable registration functionality for thoracic and extra-thoracic lesions available to clinical end users and researchers.

### Patients and tumor lesions

Patients with advanced (stages III and IV) NSCLC were studied. All underwent serial ^18^ F-FDG PET/CT prior to and after 6 weeks of chemotherapy as part of routine clinical care. No consent was necessary from the Medical Ethics Committee because of the retrospective nature of this study, under the Dutch Medical Research involving Human Subjects Act.

### ^18^ F-FDG PET/CT

^18^ F-FDG PET/CT scanning was performed on a Siemens Biograph mCT 64 slice PET/CT scanner (Siemens Healthcare, Erlangen, Germany). Blood glucose levels were consistently checked and recorded. All values were below 11 mmol/L. A weight-dependent ^18^ F-FDG dose (3 MBq/kg bodyweight) was administered to the patients intravenously, and 60 min post-injection PET/CT scan was acquired from the mid-thigh to the brain [17]. Prior to PET imaging, a low-dose CT scan was acquired craniocaudally during shallow breathing. Effective tube current was 24 mAs, tube voltage of 100 kV and care dose switched on. Slice thickness was 2 mm, and pitch 1.5 with a gantry rotation time of 0.5 s.

PET imaging was performed with a scan time per bed position dependent on patient weight. Scan time per bed position was 1 min for a patient weighing less than 60 kg, 2 min for a patient weighing between 60 and 90 kg, and 3 min for a patient weighing above 90 kg [17]. PET data were corrected for attenuation using the low-dose CT data while a delayed coincidence window was used for random correction and a model-based scatter correction was applied.

PET data were reconstructed using a transaxial image matrix of 256 × 256 and an ordered subset expectation maximization (OSEM) algorithm taking advantage of time of flight information and modeling the system response. This scheme used 3 iterations each consisting of 21 subsets. The reconstructed volumetric voxel size was 3.2 × 3.2 × 2.0 mm (20.8 mm^3^). A Gaussian filter with 8-mm full width half maximum (FWHM) was used to smooth the reconstructed data, such that recovery coefficients were in line with EANM guidelines [18].

Per patient multiple metabolically active tumor lesions were delineated on the reference and the baseline scan using the 41% adaptive thresholding technique [19]. VOI lesions were classified as both thoracic with presumed movement errors and extra-thoracic localization with minimal movement errors. Lesions were defined as thoracic if they were within the lung, mediastinum, and in or against the interior side of the thoracic wall. The largest thoracic lesion was defined as the primary tumor.

### CT-based response assessment

Contrast-enhanced CT of the thorax was performed on the Biograph mCT in the same session. Scanning was performed craniocaudally in 8 s, with breath hold at inspiration. Effective tube current was 80 mAs with tube voltage of 120 kV and care dose settings on. Slice thickness was 0.5 mm, and pitch was 1.4 with a gantry rotation time of 0.5 s. Patients were injected with 55 mL of Iomeron contrast (350 mg/mL) at a speed of 2.5 mL/s, starting 30 s before the start of the scan. Tumor response was measured on CT according to RECIST 1.1 criteria [1].

### Propagated align algorithm function

The propagated align algorithm, which is part of the PC-based IMALYTICS Research Workstation (Philips Innovative Technologies GmbH, Aachen, Germany) is based on the elastix toolbox (version 4.6), a public domain computer program for intensity-based medical image registration [15]. The elastix-based propagated align algorithm incorporates a graphical user interface and provides a cubic B-spline-based non-linear algorithm to correct for deformations. Rigid and deformable image registration is performed on the low-dose CT data, and the resulting transformations are thereafter applied on the sequentially acquired PET scans.

CT to CT registration errors were previously investigated and known to be small [16,20]. Consequently, they were not further studied in this paper.

The procedure for the deformable registration of a target PET/CT dataset to a reference PET/CT dataset is as follows:

1. The rigid transformation for optimal alignment of the target CT to the reference CT dataset is determined.
2. Subsequently, the resulting rigid transformation (translation and rotation) is applied to the target PET to ensure that the target PET properly aligns to the reference PET.
3. Next, a non-rigid transformation is performed on the rigidly aligned CT images as discussed by Klein et al. [15]. The result is a deformation vector field (Table 1) based on cubic B-splines mapping the rigidly transformed target CT onto the reference CT. For quality assurance, the Jacobian determinant (Table 1) of the deformation vector field is extracted as well, since it provides information on the local deformations (expansion/contraction). No additional constraints to the deformation field are imposed.

**Table 1:** **Alignment percentage per patient between elastix and baseline image of intra- and extra-thoracic measurable lesions**

**Pts**				**Thoracic**			**Extra-thoracic**		
	**Age**	**Sex**	**RECIST**	***N***	**% ΔSUV**	**%Δ perc.**	***N***	**% ΔSUV**	**%Δ perc.**
1.	60	F	PR	2	92	67	-		
2.	44	F	PD	1	92	99	-		
3.	55	F	SD	3	96	90	-		
4.	79	F	PR	2	99	94	-		
5.	70	F	PR	1	100	87	-		
6.	21	F	PR	3	97	95	3	100	100
7.	65	M	SD	5	57*	90	4	90	97
8.	57	F	PR	7	86	81	-		
9.	62	F	PR	1	97	91	1	100	85
10.	48	F	SD	1	92	37	-		
11.	65	M	PD	3	87	87	13	96	98
12.	49	F	SD	3	100	97	2	100	96
13.	43	M	SD	1	100	100	5	99	99
14.	65	F	PR	2	78*	76	-		
15.	51	F	SD	6	98	94	-		
16.	52	M	SD	6	100	97	-		
17.	72	F	SD	1	95	94	-		
18.	56	F	PD	3	73*	93	1	93	93
19.	71	M	PR	1	88	79	-		
20.	55	F	PR	1	78*	84	-		
21.	40	F	PR	2	100	89	6	99	94
22.	62	F	SD	2	100	95	2	100	100
23.	62	F	SD	0	**		1	100	100
24.	56	F	SD	2	100	99	-		
25.	75	M	PR	3	100	86	-		
26.	47	F	PR	1	100	92	-		
27.	54	M	PR	9	91	84	9	100	97
28.	65	M	SD	3	96	97	-		
29.	59	F	SD	5	94	97	-		
30.	72	M	SD	1	89	94	-		
31.	63	M	PR	8	96	68	-		
32.	62	M	PD	8	90	89	12	92	92
33.	36	F	PR	1	99	87	-		
34.	64	F	PD	6	100	100	4	100	100
35.	40	F	PD	4	90	80	6	99	78
36.	40	M	SD	2	96	90	5	100	96
37.	61	M	PR	1	100	88	1	100	96
38	68	M	PR	1	51*	57	-		
39.	26	F	CR	3	91	75	1	99	91
Total		14 M/25 F		115			76		
Median	59				96	90		100	96

*N*, number of measurable lesions per patient; % ΔSUV, percentage of the voxels that fall within the 95% limits of agreement of −1.46 to 1.46 ΔSUV; % Δ% perc., percentage of the voxels that fall within the 95% limits of agreement of −25 to 25 Δ percentage; CR, complete response; PR, partial response; SD: stabile disease; PD, progressive disease; −, no extra-thoracic lesions present. *Misalignment caused by adjacent normal tissue. **Patient with a solitary metastasis post curative surgical treatment. RECIST 1.1 response assessment.
4. The following step is to propagate the deformation field to the rigidly aligned PET images from step 2. In this way, target PET images are deformed to spatially match the reference PET data.

**Figure 1:**
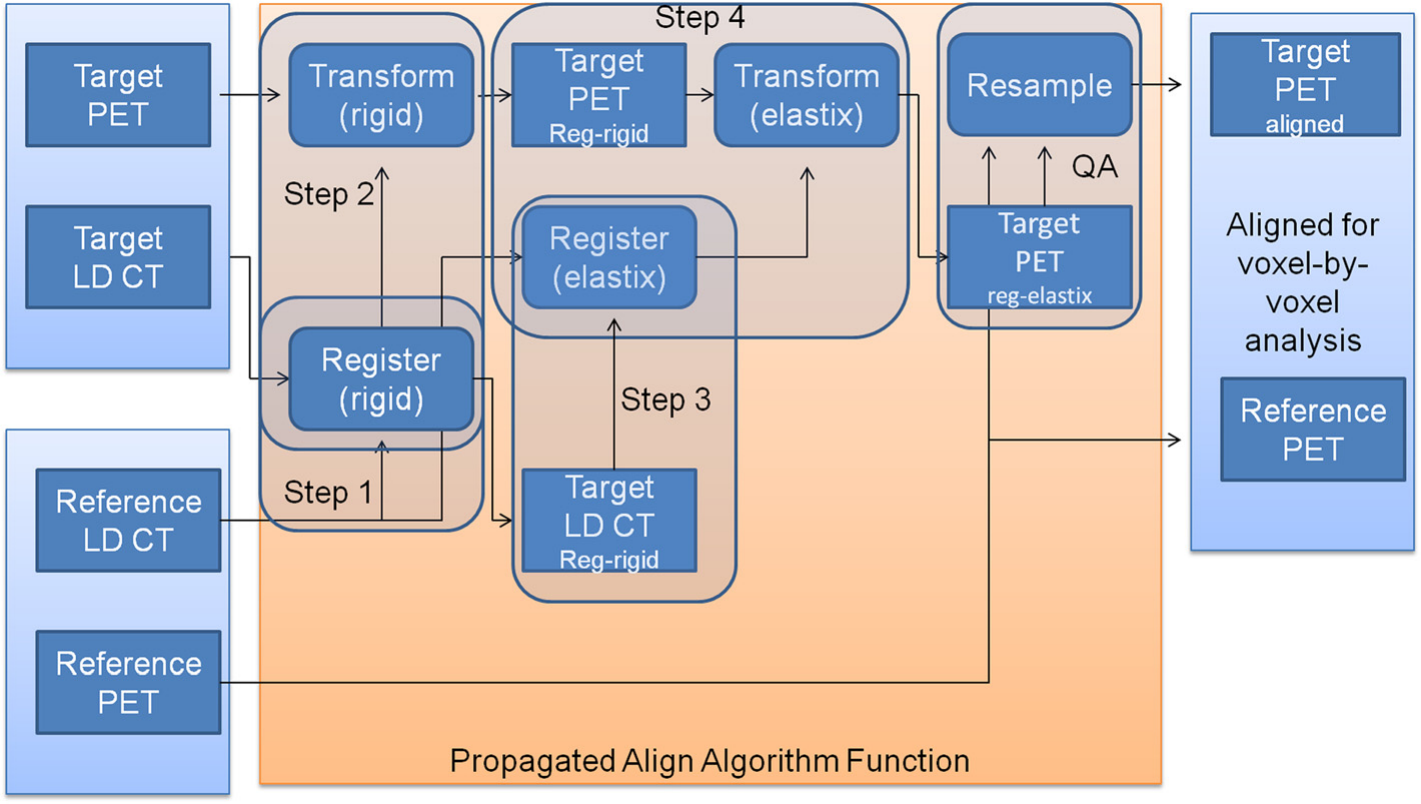
**Fusion steps of the propagated align algorithm.** Reg-rigid: rigidly registered image. Reg-elastix: rigid and non-rigidly registered image. Step 1: rigid CT to CT alignment of target to reference. Step 2: translation of step 1 on target PET. Step 3: CT to rigid aligned CT non-rigid alignment using elastix toolbox. Step 4: translation of step 3 to PET of step 2. QA: resample of image as quality assurance, so voxel size matches prior to voxel-by-voxel comparison.

The fusion steps are also detailed in Figure 1. All transformation calculations used a four-level multi-resolution approach while mutual information as image similarity measure, and an adaptive stochastic gradient descent optimizer [21] was used to maximize image similarity. The control point spacing of the B-spline transformation was 16 mm. The maximum number of iterations was set to 250 (rigid) and 500 (B-spline). Because of computation time, data were processed in batch mode using python scripting.

Quality assessment of the image registration was performed systematically by visually comparing each non-rigidly aligned CT to the reference CT scan.

### Longitudinal VOI-based and voxel-by-voxel analysis

All previous steps are necessary in order to achieve deformable image registration of PET images acquired at different time points. Next, the deformed or ‘warped’ image registration was evaluated in terms of both lesion statistics and voxel-by-voxel mapping of tumoral ^18^ F-FDG uptake.

First, the lesion statistics were investigated as follows: the follow-up PET data were transformed and interpolated to the spatial voxel locations of the baseline PET data. Tumor delineations of the baseline PET scan were used to calculate lesion statistics of both baseline and the aligned follow-up data. These findings were compared with the lesion statistics determined on the original (non-warped) follow-up PET data. This was done in order to evaluate the impact of deformed and interpolated follow-up data on therapy response assessment.

Secondly, in terms of voxel-by-voxel mapping of tumoral ^18^ F-FDG uptake between different PET time points, the original follow-up dataset was aligned to the baseline PET/CT (which was used for the lesion statistics). An elastix deformable image registration was performed again to realign the warped follow-up dataset back with the original follow-up dataset. The aim is to assess to which extent the original follow-up tumor uptake values is preserved by applying image registration using the elastix toolbox. If there were any large discrepancies (i.e., <80% alignment using ΔSUV), we further investigated the reason for this low alignment percentage.

### Impact of PET signal to noise ratio (SNR) on the voxel-by-voxel analysis

Signal to noise measurements were performed by defining VOIs in healthy pulmonary and hepatic tissue of the original PET data. A spherical VOI of 5 cm diameter was placed in the right lobe of the liver, while a 3-cm-diameter spherical VOI was positioned in healthy lung tissue.

### Statistics

The correlation coefficient between original and realigned tumor uptake values (SUV) was determined using Pearson’s *R* test. The difference between original and realigned tumor uptake values was calculated both as the difference in standard uptake values (ΔSUV) and the difference in percentage of the original SUV (Δ percentage). Equivalence between original and realigned follow-up tumor uptake values was assessed by a Bland-Altman analysis with the original follow-up tumor data used as reference method. For the Bland-Altman analysis, ΔSUV and Δ% SUV corresponding to the 95% limits of agreement was determined taking into account the tumor voxels of all measurable tumor lesions in all patients.

To assess the influence of spatial discrepancies between PET and CT data due to respiratory and cardiac motion, equivalence between original and realigned tumor uptake values was assessed per patient separately for thoracic and extra-thoracic lesions. The significance of the equivalence between thoracic and extra-thoracic lesions was assessed using a chi-square test.

To assess the effects of SNR, the mean activity of the lung and liver spherical VOI were divided by the standard deviation (SD) of the activity. This was performed to estimate for the signal to noise ratio. Impact of the PET SNR on the voxel-by-voxel statistical analysis was evaluated by assessing the relationship between PET SNR and equivalence between original and realigned tumor uptake values.

All statistical analyses were performed using SPSS version 20.0 (International Business Machines Corp, Armonk, NY, USA). Nominal *P* values less than 0.05 were considered significant.

## Results

A total of 39 patients were evaluated. There were 37 patients with stage IV disease and 2 with stage III. Median age was 59 (21 to 79) years, and male/female ratio was 14/25. Body weight changed less than 1 kg during the study.

### Delineated tumor lesions

For each of the 39 patients, a median (range) number of 3 (1 to 20) VOIs were delineated on the original baseline fluorodeoxyglucose (FDG) PET scan with per tumor VOI localization. The range of the volume of the primary tumor was 2.4 to 556.8 mL (median 25.5 mL), and for the metastasis, it was 1.2 to 390 mL (median 7.8 mL). For the 39 patients, a total of 191 different tumors (primaries and metastases) consisting of 295,219 tumor voxels (6,141 mL) were considered.

### Equivalence of the original and realigned baseline tumor uptake values

On visual assessment, all target CT aligned properly to the respective reference CT. There was a good relationship between the voxel values of the original and elastix adjusted images (Pearson correlation test; *R*^2^ 0.98; *P* < 0.01).

#### Equivalence testing using ΔSUV

**Figure 2:**
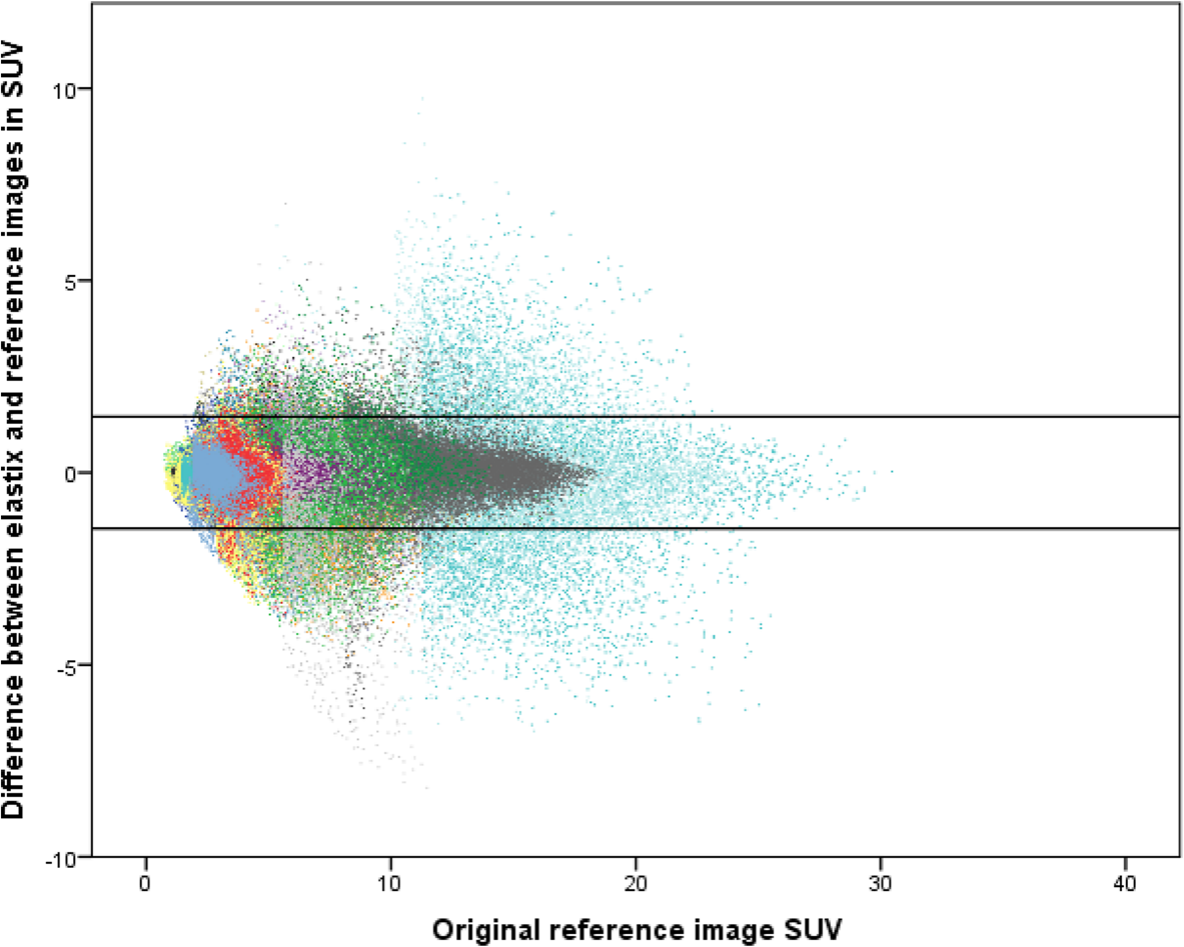
**Equivalence plot between reference and realigned images of 39 patients.** 94% of the voxels are within the 95% range of the difference.

The SD of the ΔSUV between reference and target images was 0.74. With the range of the 95% limits of agreement defined as between −1.46 and 1.46 ΔSUV, a total of 94% of all voxels was accurately aligned within the range, or in other words, 6% of the voxels mismatched (Figure 2). Using ΔSUV, 34 patients had a thoracic alignment (i.e., the voxel percentage with an alignment within the 95% estimated range) of above 80%. Five patients had an alignment less than 80% (Table 1; see below). The extra-thoracic locations aligned above 90% of the voxels in all patients implicating that movement control was adequate (Table 1).

**Figure 3:**
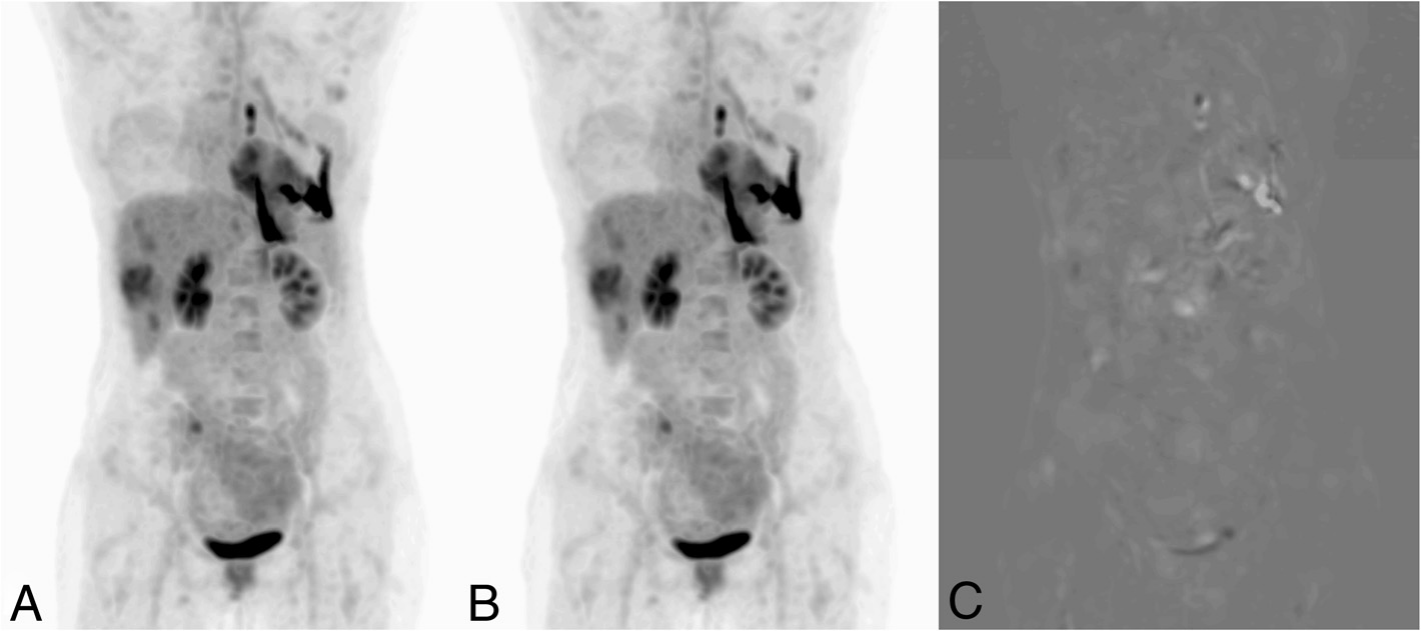
**Original (A), elastix adjusted (B), and subtraction(C) maximum intensity projection of patient 6.** These images show the typical results before and after alignment as well as the difference between these two images.

A typical example of a patient is shown in Figure 3, with the reference, target and target minus reference image.

**Figure 4:**
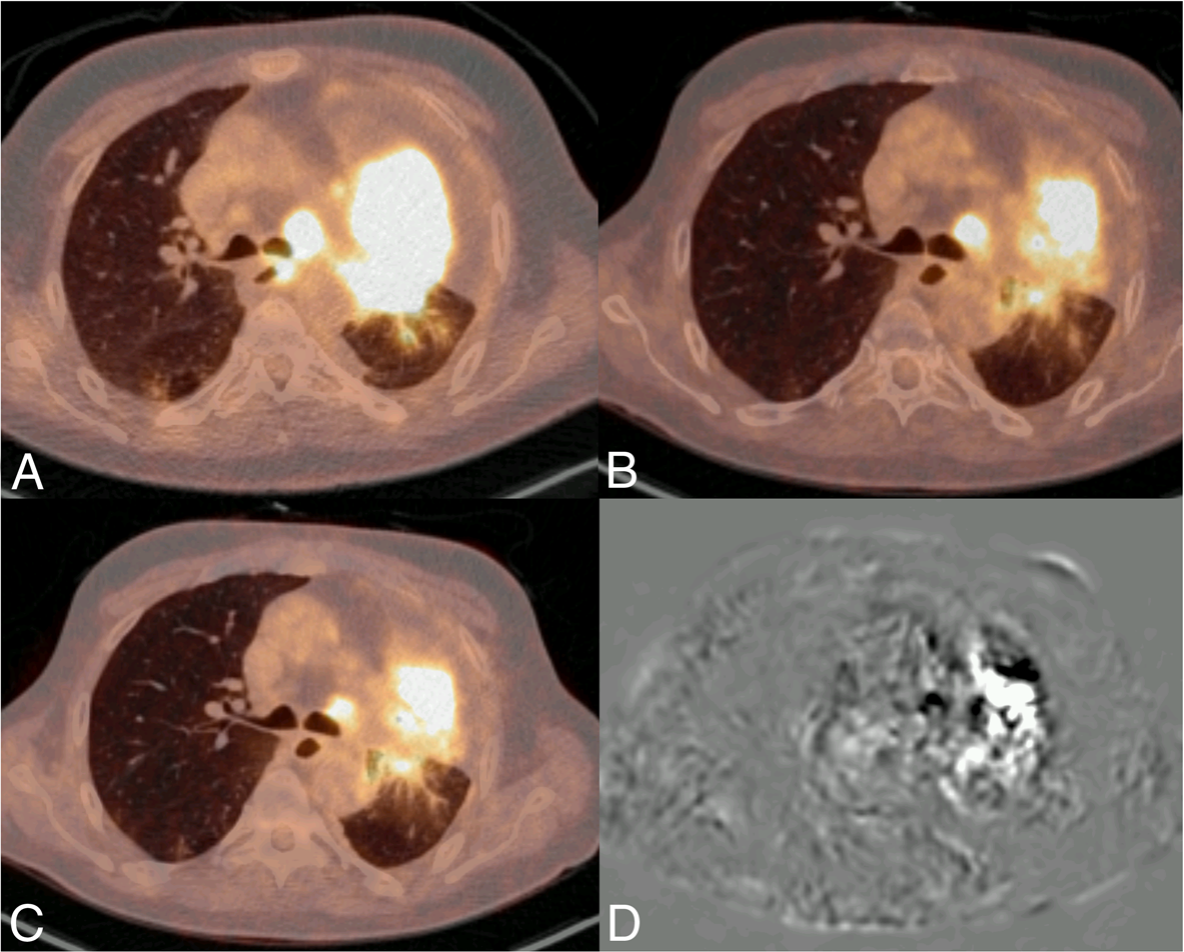
**Example image of patient 7: baseline (A), reference (B), elastix adjusted (C) and subtraction (D).** Because of a mix of non-active and active tumor tissue between the two scans (and no visible decrease in anatomic tumor size with stable disease), only 57% of the voxels aligned within the 95% limits of agreement of −1.46 to 1.46 ΔSUV. However, on crude visual assessment, the images look very similar.

Further study of the 5 patients with a voxel alignment <80% revealed that the reason for misalignment in general was due to CT- to PET-specific-related inaccuracies. In some patients, these inaccuracies were specific to therapy as it involved tumor necrosis (Figure 4). The other reason for an inaccuracy was the subsequent presence or absence of collapsed lung tissue after therapy (Additional file 1: Table S1).

#### Equivalence testing using Δ percentage

The SD of the Δ percentage between reference and target images was 13%. With the range of the 95% limits of agreement defined as between −25 and 25 Δ percentage, a total of 93% of voxels aligned within this range, which means that 7% of the voxels mismatched using this method. The alignment percentage per patient using ΔSUV and Δ percentage is detailed in Table 2.

**Table 2:** **SUV**_**max**_ **of thoracic and non-thoracic lesions and the difference between original and elastix adjusted image**

**Pts**		**Thoracic**			**Extra-thoracic**		
	**RECIST**	**Original**	**Elastix**	**Difference**	**Original**	**Elastix**	**Difference**
1.	PR	5.5	6.5	1.0			
2.	PD	18.2	18.2	0.0			
3.	SD	6.9	7.3	0.4			
4.	PR	6.4	7.1	0.7			
5.	PR	3.8	4.0	0.2			
6.	PR	11.2	10.8	−0.4	4.6	3.1	−1.4
7.	SD	25.5	30.8	5.3			
8.	PR	10.7	10.7	0			
9.	PR	5.9	6.5	0.6	4.2	4.5	0.3
10.	SD	4.1	3.6	−0.5			
11.	PD	13.6	9.8	−3.8	14.5	10.2	−4.3
12.	SD	7.2	6.8	−0.4	6.5	3.1	−3.4
13.	SD	1.8	1.7	−0.1	12.3	11.4	−0.9
14.	PR	10.8	12.3	1.5			
15.	SD	7.7	6.6	−1.1			
16.	SD	7.0	6.4	−0.6			
17.	SD	10.1	9.3	−0.8			
18.	PD	24.2	31.0	6.8			
19.	PR	6.3	4.5	−1.8			
20.	PR	12.0	12.0	0			
21.	PR	4.7	6.3	1.6	7.0	9.1	2.1
22.	SD	6.8	6.8	0.0	3.4	2.3	−1.2
23.	SD	*			5.6	4.7	−0.9
24.	SD	7.2	7.0	−0.3			
25.	PR	3.9	4.2	0.3			
26.	PR	3.0	2.9	0.1			
27.	PR	6.5	8.2	1.7	2.9	6.1	3.1
28.	SD	14.1	9.0	−5.2			
29.	SD	18.8	18.3	−0.5			
30.	SD	14.8	15.4	0.7			
31.	PR	5.9	6.5	0.6			
32.	PD	*			9.8	11.4	1.7
33.	PR	5.0	4.8	−0.2			
34.	PD	11.4	11.7	0.3	7.4	7.6	0.2
35.	PD	6.3	5.3	−1.0	2.6	2.3	−0.3
36.	SD	6.3	4.1	−2.2			
37.	PR	4.1	4.2	0.1			
38	PR	13.2	13.3	0.1			
39.	CR	4.0	5.1	1.1			

*No thoracic lesion measurable, RECIST 1.1 response assessment. CR, complete response; PR, partial response; SD, stabile disease; PD, progressive disease.

**Figure 5:**
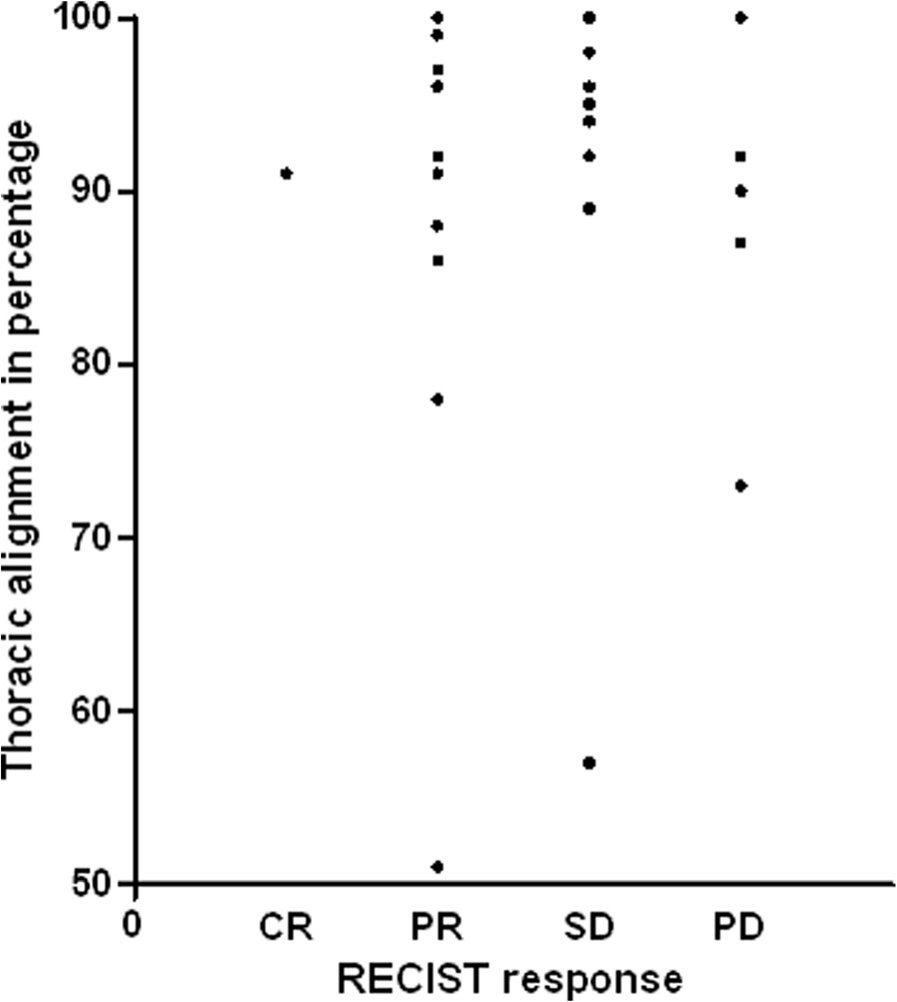
**Relation between thoracic imaging alignment and tumor response according to RECIST criteria per patient.** RECIST tumor response was not related to the thoracic alignment percentage.

There was no relation between the alignment percentage and tumor response (Figure 5), signal to noise ratio of either the liver or normal parts of the lung, or with the distribution of the original SUV.

### Assessment of effects on SUV_max_

It was not possible to perform simultaneous measurement of the SUV_max_ in two patients. One (nr. 23) had only a solitary extra-thoracic metastasis. The other patient (nr. 32) had at baseline no measurable thoracic lesions; however, there was a significant amount of measurable lesions after 6 weeks of therapy. The median difference of SUV_max_ of the primary tumor between the elastix adjusted to baseline image and the original image was 0.0 SUV (−5.2 to 6.8 SUV), while for extra-thoracic lesions it was −0.6 SUV (−4.3 to 3.1 SUV). When measured using the EORTC criteria, concerning the primary tumor, 5/37 patients had a discordant response, whereas in the extra-thoracic lesions, 2/12 patients had a discordant response (Table 2).

## Discussion

In this study, we investigated whether a non-linear realignment of PET images using the elastix toolbox can be used for therapy assessment on patients with NSCLC. This allows the reuse of baseline lesion delineations for the quantification of tracer uptake in tumor lesions at follow-up. Also, it may facilitate therapy response assessment using EORTC or PERCIST criteria. Furthermore, this approach may allow an automated volumetric voxel-by-voxel analysis as it delivers more information on tumor changes. Changes in the distribution of voxels under the influence of treatment in terms of percentage viable voxels (i.e., percentage above or below a predetermined cutoff point) within a certain volume of interest may be a better indicator of efficacy and consequently have prognostic value. In order to make this method feasible, a reliable alignment technique for PET scans is paramount. We used the elastix toolbox which had shown good performance in different CT-based thoracic alignment studies [22,23]. Anatomical information such as lung boundaries and major lung fissures and correspondence to annotated landmark pairs were used, and the results showed a good performance. The toolbox makes use of a cubic B-spline non-linear algorithm which shows good mass preservation between inspiration and expiration CT scans [24]. Our study not only used the CT-based alignment of the elastix toolbox but also projected this alignment on the corresponding ^18^ F-FDG PET images, and the performance was assessed using both lesion statistics and voxel-by-voxel analysis.

Our results show that the method has significant impact on lesion statistics. This impact, with differences between −5 and 7 ΔSUV in thoracic lesions, is clinically relevant. First of all, such large spread of 12 SUV affects the interpretation of the results in a clinically significant way. Secondly, we observed changes in the response assessment in 5 out of 37 (14%) patients using the 1999 EORTC response proposal. The elastix toolbox settings were optimized for CT-CT registration where the alignment of anatomical structures in the lungs was the primary target. For longitudinal PET quantification, other settings might be more appropriate. On the other hand, the approach for volumetric tumor analysis used by van Velden et al. where instead of interpolating follow-up data to match baseline voxel locations, the baseline VOIs are projected onto the follow-up data, may be considered more appropriate for longitudinal tracer quantification [25]. The reason that it might be more appropriate is that this approach does not allow a voxel-by-voxel analysis but allows a more accurate quantification of global tumor characteristics such as SUV_max_.

The second goal of using deformable image registration and realigning follow-up with baseline data was to be able to measure more accurately intratumoral heterogeneity (textural analysis) and response to treatment in a more detailed manner. This is important, as initially measured high intratumoral heterogeneity on ^18^ F-FDG PET is related both to poor prognosis and to resistance to treatment [26]. We demonstrate that the original intratumoral tracer distribution indeed can be preserved by using a forward and backward deformable image registration. This means that the registration process is nearly invertible and information about intratumoral tracer distribution is preserved to a great extent during the registration.

The propagated align algorithm itself is a shell that makes the multiple steps necessary for performing deformable image registration using the elastix toolbox more user friendly. It provides an automated method to perform not just the multiple steps per patient but also the multiple patients per instance. It can also be adapted to other deformable image registration methods.

### Methodological shortcomings

There are some factors that negatively influence the application of this technique.

First, in 5 patients, there was a voxel misalignment in the thorax, which was caused by the presence of mediastinal tissue directly adjacent to the tumor tissue. We need to consider that the first step of the alignment method is based on anatomical CT changes and that on CT, hardly no difference can be made between the collapsed lung, tumor necrosis, or mediastinal tissue, whereas PET can do so. Consequently, in these cases, the method will introduce discrepancies, that will need further attention.

Second, as was seen with the SUV_max_ measurement, in some patients, the SUV_max_ was different with a widespread on the elastix adjusted image and the original image. This effect was particularly pronounced in thoracic lesions. However, because the voxel-by-voxel analysis showed that values correspond well after forward and backward transformations, it suggests that the corresponding transformation is practically invertible. This is a concern that needs further optimization of the algorithm and limits the applicability of the algorithm in its current stage. This problem could be exacerbated due to the use of 3D instead of 4D PET. A previous study showed significant differences in textural features between static 3D and respiratory-gated 4D PET/CT [27]. The same study showed higher uptake and less blurring in the 4D PET images compared to the corresponding 3D PET images [27].

Third, we were not able to further compare the textural features between the images. Textural features can predict disease recurrence and survival, sometimes more powerfully than the current global measurements used in clinical practice [11,26]. This should be the subject of future studies.

## Conclusions

Comparative imaging analyses with an automated voxel-by-voxel technique are a promising tool for tumor evaluation in patients with advanced NSCLC. However, the elastix toolbox impacts lesion statistics and therefore therapy response assessment in a clinically significant way. Consequently, the elastix toolbox is not applicable in its current form and/or standard settings for response evaluation. It remains to be determined to which extent the intratumoral uptake distribution is preserved when this type of deformable image registration is applied. Further optimization and validation is necessary due to the inaccuracies observed in this study. The propagated align algorithm provides the shell for performing deformable image registration, in this case, using the elastix toolbox. The propagated align algorithm itself could be adapted for performing deformable image registration using other techniques.

## Additional files

Additional file 1: Table S1. Reason for less than 80% alignment using ΔSUV.

## References

[1] Eisenhauer EA, Therasse P, Bogaerts J, Schwartz LH, Sargent D, Ford R (2009). New response evaluation criteria in solid tumours: revised RECIST guideline (version 1.1). Eur J Cancer..

[2] Sunaga N, Oriuchi N, Kaira K, Yanagitani N, Tomizawa Y, Hisada T (2008). Usefulness of FDG-PET for early prediction of the response to gefitinib in non-small cell lung cancer. Lung Cancer..

[3] Kobe C, Scheffler M, Holstein A, Zander T, Nogova L, Lammertsma AA (2012). Predictive value of early and late residual 18 F-fluorodeoxyglucose and 18 F-fluorothymidine uptake using different SUV measurements in patients with non-small-cell lung cancer treated with erlotinib. Eur J Nucl Med Mol Imaging..

[4] Tiseo M, Ippolito M, Scarlattei M, Spadaro P, Cosentino S, Latteri F (2014). Predictive and prognostic value of early response assessment using 18FDG-PET in advanced non-small cell lung cancer patients treated with erlotinib. Cancer Chemother Pharmacol..

[5] Young H, Baum R, Cremerius U, Herholz K, Hoekstra O, Lammertsma AA (1999). Measurement of clinical and subclinical tumour response using [18 F]-fluorodeoxyglucose and positron emission tomography: review and 1999 EORTC recommendations. European Organization for Research and Treatment of Cancer (EORTC) PET Study Group. Eur J Cancer.

[6] Wahl RL, Jacene H, Kasamon Y, Lodge MA (2009). From RECIST to PERCIST: evolving considerations for PET response criteria in solid tumors. J Nucl Med..

[7] Cerfolio RJ, Bryant AS, Ohja B, Bartolucci AA (2005). The maximum standardized uptake values on positron emission tomography of a non-small cell lung cancer predict stage, recurrence, and survival. J Thorac Cardiovasc Surg..

[8] Paesmans M, Berghmans T, Dusart M, Garcia C, Hossein-Foucher C, Lafitte JJ (2010). Primary tumor standardized uptake value measured on fluorodeoxyglucose positron emission tomography is of prognostic value for survival in non-small cell lung cancer: update of a systematic review and meta-analysis by the European Lung Cancer Working Party for the International Association for the Study of Lung Cancer Staging Project. J Thorac Oncol..

[9] Bille A, Okiror L, Skanjeti A, Errico L, Arena V, Penna D (2013). The prognostic significance of maximum standardized uptake value of primary tumor in surgically treated non-small-cell lung cancer patients: analysis of 413 cases. Clin Lung Cancer..

[10] de Jong WK, van der Heijden HF, Pruim J, Dalesio O, Oyen WJ, Groen HJ (2007). Prognostic value of different metabolic measurements with fluorine-18 fluorodeoxyglucose positron emission tomography in resectable non-small cell lung cancer: a two-center study. J Thorac Oncol..

[11] Cook GJ, Yip C, Siddique M, Goh V, Chicklore S, Roy A (2013). Are pretreatment 18 F-FDG PET tumor textural features in non-small cell lung cancer associated with response and survival after chemoradiotherapy?. J Nucl Med..

[12] Ma B, Meyer CR, Pickles MD, Chenevert TL, Bland PH, Galban CJ (2009). Voxel-by-voxel functional diffusion mapping for early evaluation of breast cancer treatment. Inf Process Med Imaging..

[13] Galban CJ, Chenevert TL, Meyer CR, Tsien C, Lawrence TS, Hamstra DA (2009). The parametric response map is an imaging biomarker for early cancer treatment outcome. Nat Med..

[14] Schreibmann E, Waller AF, Crocker I, Curran W, Fox T (2013). Voxel clustering for quantifying PET-based treatment response assessment. Med Phys..

[15] Klein S, Staring M, Murphy K, Viergever MA, Pluim JP (2010). Elastix: a toolbox for intensity-based medical image registration. IEEE Trans Med Imaging..

[16] Murphy K, van Ginneken B, Reinhardt JM, Kabus S, Ding K, Deng X (2011). Evaluation of registration methods on thoracic CT: the EMPIRE10 challenge. IEEE Trans Med Imaging..

[17] de Groot EH, Post N, Boellaard R, Wagenaar NR, Willemsen AT, van Dalen JA (2013). Optimized dose regimen for whole-body FDG-PET imaging. EJNMMI Res..

[18] Boellaard R, O’Doherty MJ, Weber WA, Mottaghy FM, Lonsdale MN, Stroobants SG (2010). FDG PET and PET/CT: EANM procedure guidelines for tumour PET imaging: version 1.0. Eur J Nucl Med Mol Imaging..

[19] Cheebsumon P, Boellaard R, de Ruysscher D, van Elmpt W, van Baardwijk A, Yaqub M (2012). Assessment of tumour size in PET/CT lung cancer studies: PET- and CT-based methods compared to pathology. EJNMMI Res..

[20] Kanai T, Kadoya N, Ito K, Onozato Y, Cho SY, Kishi K (2014). Evaluation of accuracy of B-spline transformation-based deformable image registration with different parameter settings for thoracic images. J Radiat Res..

[21] Klein S, Pluim JPW, Staring M, Viergever MA (2009). Adaptive stochastic gradient descent optimisation for image registration. Int J Comput Vis..

[22] Staring M, Klein S, Reiber JHC, Niessen WJ, Stoel BC (2010). Pulmonary image registration with elastix using a standard intensity-based algorithm. Book pulmonary image registration with elastix using a standard intensity-based algorithm.

[23] Kabus S, Klinder T, Murphy K, van Ginneken B, van Lorenz C, Pluim JP (2009). Evaluation of 4D-CT lung registration. Med Image Comput Comput Assist Interv..

[24] Yin Y, Hoffman EA, Lin CL (2009). Mass preserving nonrigid registration of CT lung images using cubic B-spline. Med Phys..

[25] van Velden FH, van Beers P, Nuyts J, Velasquez LM, Hayes W, Lammertsma AA (2012). Effects of rigid and non-rigid image registration on test-retest variability of quantitative [18 F]FDG PET/CT studies. EJNMMI Res..

[26] Chicklore S, Goh V, Siddique M, Roy A, Marsden PK, Cook GJ (2013). Quantifying tumour heterogeneity in 18 F-FDG PET/CT imaging by texture analysis. Eur J Nucl Med Mol Imaging..

[27] Yip S, McCall K, Aristophanous M, Chen AB, Aerts HJ, Berbeco R (2014). Comparison of texture features derived from static and respiratory-gated PET Images in non-small cell lung cancer. PLoS One..

