# Supplementary material for: Evaluation of elastix-based propagated align algorithm for VOI- and voxel-based analysis of longitudinal 18F-FDG PET/CT data from patients with non-small cell lung cancer (NSCLC)
Source: EJNMMI Res. 2015 Mar 21;5:15. doi: 10.1186/s13550-015-0089-z (PMC4385310; doi:10.1186/s13550-015-0089-z)
Supplement: Additional file 1: Table S1. — Reason for less than 80% alignment using ΔSUV. [file 13550_2015_89_MOESM1_ESM.doc]

Additional file 1: Table S1: reason for less than 80% alignment using Δ SUV

_______________________________________________________________________________

Patient 7: Significant decrease in activity after therapy within remaining collapsed lung.

Patient 14: Significant decrease of collapsed lung adjacent to tumor tissue

Patient 18: Adjacent to upper mediastinum, central tumor necrosis after treatment

Patient 20: Decreased tumor mass, however increase in pleural fluid

Patient 38: Central tumor necrosis post treatment

_______________________________________________________________________________
